# Systemic treatment with cigarette smoke extract affects zebrafish visual behaviour, intraocular vasculature morphology and outer segment phagocytosis

**DOI:** 10.12688/openreseurope.15491.2

**Published:** 2023-11-23

**Authors:** Alicia Gómez Sánchez, Patrizia Colucci, Ailis Moran, Alexandro Moya López, Basilio Colligris, Yolanda Álvarez, Breandán N. Kennedy

**Affiliations:** 1 UCD Conway Institute of Biomolecular and Biomedical Research, University College Dublin, Dublin, D04 V1W8, Ireland; 2 Ocupharm Diagnostic Group Research, Faculty of Optic and Optometry, Complutense University of Madrid, Madrid, Spain; 3 Department of Biotechnology, Chemistry and Pharmacy, University of Siena, Siena, Italy; 4 UCD School of Biomolecular and Biomedical Science, University College Dublin, Dublin, D04 V1W8, Ireland

**Keywords:** Smoking, visual behaviour, zebrafish, retina, oxidative stress

## Abstract

**Introduction:**

Cigarette smoking adversely affects multiple aspects of human health including eye disorders such as age-related macular degeneration, cataracts and dry eye disease. However, there remains a knowledge gap in how constituents of cigarette smoke affect vision and retinal biology. We used zebrafish to assess effects of short-term acute exposure to cigarette smoke extract (CSE) on visual behaviour and retinal biology.

**Methods:**

Zebrafish larvae with a developed visual system at three days post-fertilization (dpf) were exposed to CSE for 4, 24 or 48 hours. Visual behaviour, hyaloid vasculature morphology, retinal histology, oxidative stress gene expression and outer segment phagocytosis were investigated using visual behavioural optokinetic and visual motor response assays (OKR and VMR), microscopy (light, fluorescence and transmission electron microscopy), and real-time PCR.

**Results:**

In zebrafish larvae, 48 hours of CSE treatment resulted in significantly reduced visual behaviour. Larvae treated with 10, 15 or 20 μg/mL CSE showed an average of 13.7, 10.7 or 9.4 saccades per minute, respectively, significantly lower compared with 0.05% DMSO controls (p=0.0093, p=0.0004 and p<0.0001, respectively) that exhibited 19.7 saccades per minute. The diameter of intraocular vessels increased from 4.833 μm in 0.05% DMSO controls to 5.885 μm in the 20 μg/mL CSE-treated larvae (p=0.0333). Biometry analysis highlighted a significant axial length elongation in 20 μg/mL CSE-treated larvae (216.9 μm, p<0.0001) compared to 0.05% dimethyl sulfoxide (DMSO) controls (205.1 μm). Larvae exposed to 20 μg/mL CSE had significantly (p=0.0002) higher numbers of RPE phagosomes compared to vehicle controls (0.1425 and 0.093 phagosomes/μm RPE, respectively).

**Conclusions:**

Zebrafish larvae with a developed visual system display apparent defects in visual behaviour and retinal biology after acute exposure to CSE, establishing a valuable *in vivo* model to investigate ocular disorders related to cigarette smoke.

## Plain language summary

This study investigates the effects of cigarette smoke on the visual system of zebrafish larvae. We exposed the larvae to cigarette smoke extract for 4, 24, or 48 hours and assessed their eye movements, retina morphology and oxidative stress gene expression. Exposure to cigarette smoke extract for 48 hours reduced eye movements behaviour in the zebrafish larvae and led to changes in the morphology of their hyaloid vasculature present in the lens and the number of phagosomes in their retinal pigment epithelium. When exposure was shortened to 4 or 24 hours, eye movements were still reduced and oxidative stress was affected. These results suggest that zebrafish larvae can be used as a valuable model to investigate ocular disorders related to cigarette smoke.

## Abbreviations

CSE: Cigarette Smoke Extract

Dpf: Days post-fertilization

OKR: Optokinetic Response

VMR: Visual Motor Response

PCR: Polymerase Chain Reaction

RPE: Retinal Pigment Epithelium

TPM: Total Particulate Matter

PaHs: Polycyclic Aromatic Hydrocarbons

IARC: International Agency for Research on Cancer

AMD: Age-related Macular Degeneration

Hpf: Hours post-fertilization

AREC: Animal Research Ethics Committee

Wt: wild type

EGPF: Enhanced Green Fluorescent Protein

FTC: Federal Trade Commission Smoke 

DMSO: Dimethyl sulfoxide

MTD: Maximum Tolerate Dose

VA: Visual Acuity

CS: Contrast Sensitivity

RGB: Red Green Blue

Cpd: Cycles per degree

Ms/s: milliseconds per second

PFA: paraformaldehyde

TEM: Transmission electron microscopy

Cat: Catalase

Gpx1a: Glutathione Peroxidase 1

Casp3a: Caspase 3

Nfe2l2a: nuclear factor erythroid 2–related factor 2

ROS: Reactive Oxygen Species

SD: Standard Deviation

FP: Front Plane

OA: Optic axis

AL: Axial Length

## Introduction

Tobacco and smoking are global concerns due to their health, societal and economic impact ^1, 2^. Second-hand smoke from cigarettes is a complex mixture of mainstream smoke (smoke inhaled and exhaled by smokers) and sidestream smoke (smoke directly from the burning cigarette) for which even brief exposure can be harmful to health ^3– 5^. Sidestream smoke contains a mixture of more than 4000 chemicals ^6^. This mixture is constituted by total particulate matter (TPM) which includes solid chemicals ( *e.g.* alkaloids, polycyclic aromatic hydrocarbons also known as PaHs and N-nitrosamines) as well as gaseous components ( *e.g.* carbon dioxide, carbon monoxide, nicotine, acetaldehyde, hydrocarbons, nitrogen, ammonia, nitrosamines and hydrogen cyanide) ^7^. Second-hand smoke is a Group 1 carcinogen, the highest carcinogenic level of the International Agency for Research on Cancer (IARC) ^4, 8^. In 2019, smoking and second-hand smoke were directly related to 7.69 and 1.2 million deaths worldwide, respectively ^9, 10^.

Smoking is well-known to promote carcinogenicity, induce developmental toxicity ^5, 11^ and cardiovascular effects ^11, 12^. Second-hand smoke is a strong risk factor for developing dry or neovascular types of age-related macular degeneration (AMD) ^3, 13, 14^; which is the leading cause of severe visual impairment in Europe ^6, 15^. Smokers and passive smokers are also at higher risk of nuclear cataracts and dry eye disease ^13^.

The use of zebrafish as an animal model to identify environmental substances impairing vision has increased considerably in recent years ^16, 17^. Advantages of zebrafish include the similarity in eye structure with the human eye, accessibility of the model to handling and manipulation, the ability to efficiently perform systemic drug administration and rapid advances in visual behaviour assays ^16, 18– 20^.

Recent studies investigating toxic effects of CSE in zebrafish identified lethality, abnormal swimming behaviour ^21^, hyperactivity ^22, 23^ and cardiovascular anomalies ^24^. Ellis *et al.* reported eye malformation in zebrafish exposed to CSE from 6 to 72 hours post-fertilization (hpf) ^21^. However, there are no studies investigating the impact of CSE on zebrafish visual behaviour, despite previous studies showing CSE exerts oculotoxic effects in mice ^25– 27^ and RPE cells ^28^. Putative research models of AMD were reported based on exposing mice to an experimental chamber for six months where cigarettes were artificially smoked ^27^. This study showed CSE to trigger oxidative damage, cell degeneration and cell apoptosis in the RPE, as well as signs of drusen formation ^27^. In another study, in which mice were exposed to CSE under similar conditions but for a shorter period of time (∼3.7 months), sub-RPE deposits and thickening of Bruch's membrane were observed ^26^. Mice exposed to CSE for 12 weeks were reported to also show apoptosis of both corneal and conjunctival epithelium cells leading to dry eye, potentially induced by inflammation ^25^. In summary, CSE exposure can adversely impact the eye and visual system, outcomes that warrant further investigation. Here we apply zebrafish as a vertebrate animal model to explore the effects of cigarette smoke on visual behaviour and retinal biology.

## Methods

### Zebrafish maintenance

All experiments using animals were carried out at University College Dublin and approved by the University College Dublin Animal Research Ethics Committee (AREC). All possible efforts were undertaken to minimise the suffering of animals. Adult zebrafish ( *Danio Rerio*) WT-Tü *(wt)* and transgenic line Tg( *fli1:eGFP)* were maintained on a recirculating water system at 28°C and pH 7.1 on a 14:10 h light-dark cycle. Water conductivity averaged 1347 µS. Adult zebrafish were fed shrimp and dry pellet food twice daily. Following afternoon feeds, male and female adults were placed in breeding tanks. Embryos were obtained by natural spawning, collected the next morning and raised in embryo medium (0.137 M NaCl, 5.4 mM KCl, 5.5 mM Na _2_HPO _4_, 0.44 mM KH _2_PO _4_, 1.3 mM CaCl _2_, 1.0 mM MgSO _4_ and 4.2 mM NaHCO _3_ with 1 mL methylene blue) until 5 dpf ^29^.

### Cigarette smoke extract (CSE)

CSE was sourced from Murty Pharmaceuticals (US) where 3R4F Standard Research Cigarettes were smoked using a Federal Trade Commission (FTC) smoke machine under an ISO smoke regime, which takes one puff every two seconds and 35 mm of volume ^4, 30^. The resulting smoke extracted directly from the cigarette was collected by a Cambridge pad filter, into total particulate matter (TPM), and dissolved in DMSO at 40 µg/mL TPM per vial. Concentrated CSE stocks were diluted before each experiment to achieve desired concentrations.

### Exposure of zebrafish larvae to CSE

CSE exposure was assessed for durations of 4, 24 or 48 h in larvae. For 48 hours exposure, CSE was added at 72 hpf, when embryos were placed in 6 cm Petri dishes. CSE doses were added directly into the embryo medium and then refreshed at 96 hpf. Medium was replaced with fresh embryo medium at 120 hpf to stop the exposure. Dose response assays identified the maximum tolerated dose (MTD) that lead to lethality, defined as larvae lacking a heartbeat and unresponsive to touch at <131 hpf. For CSE exposure of 4 and 24 h, 72 hpf embryos were placed in 6 cm Petri dishes and a CSE dose of 20 µg/mL was added directly into the embryo medium at 96 hpf. CSE exposure was stopped at 100 hpf and 120 hpf for 4 and 24 h treatments respectively. Wild-type zebrafish larvae treated with 0.05% DMSO were used as negative controls.

### Visual behaviour, visual acuity and contrast sensitivity assays

Optokinetic response (OKR), visual acuity (VA) and contrast sensitivity (CS) assays were performed as described previously ^19^. Briefly, ≤131 hpf larvae were immobilized in 9% methylcellulose and placed in the centre of a rotating drum. The drum was rotated at 18 rpm for 30 seconds clockwise and 30 seconds anti-clockwise. Saccades per minute were counted manually. For standard OKR, 0.02 cycles per degree (cpd) was used. For VA, 0.02 cpd, 0.06 cpd and 0.2 cpd 3D-printed drums were used. For CS, 0.02 cpd 2D-printed drums with a 100% and 20% black contrast were used. VA and CS assays were done separately (using different biological replicates). For VA assays, each larva from each group was tested with the 0.02, 0.06 and 0.2 cpd drums sequentially. For CS assays, each larva from each group was tested with the 100% and then 20% black-white contrast drums sequentially ^19^.

### Visual behaviour assays with coloured OKR drums

Coloured 0.02 cpd drums were designed with alternating Black-Red, Black-Green or Black-Blue stripes (named here RGB OKR drums) ( Figure 3A). 3D-printed drums were manufactured by Materialise UK and printed with 3D printing technology in polylactic acid. The RGB codes used to 3D print the drums were as follows: RGB (255, 0, 0) for the black-red drum; RGB (0, 255, 0) for the black-green drum and RGB (0, 0, 255) for the black-blue drum. To reduce the number of larvae used for experiments and to profile responses from each larvae to all drums, we followed previously described Protocol I ^19^, *i.e.* each larva from each group was subjected sequentially to standard Black-White, then the Black-Red, Black-Green and finally to the Black-Blue drums. Groups were assessed in the following order: control, 0.05% DMSO, 10, 15 and 20 µg/mL. Biological replicate experiments were run on separate days.

### Locomotor and cardiovascular physiological assays

By gently touching the caudal fin with a pipette tip, the escape or startle response of 16 larvae in a 96 well plate was examined and manually recorded using an Olympus SZX16 fluorescent microscope. This locomotor response was classified into normal, reduced or absent ^16^. For heartbeat analysis, larvae were placed into methylcellulose followed by a 10-second desensitization period. Heart rate was observed using a Nikon SMZ800 microscope and manually counted for 30 seconds. The number of beats was multiplied by 2 to calculate the heart rate per minute.

### Visual motor response assays

To measure the visual motor response (VMR), we followed the protocol previously described by Deeti *et al.* ^16^. Individual larvae were placed in a 96-well plate and immersed in 600 μL of embryo medium. A total of 12 larvae were used per treatment group. The plate was placed in the Zebrabox recording chamber (Viewpoint Life Sciences, France) to record larval locomotor activity in response to changes in light intensity for a period of 1 h and 40 min. After 30 min of adaptation to the new environment, the light intensity changed from ON to OFF (and from OFF to ON) in 20-min intervals. Locomotor activity data were exported to MS Excel and analysed using GraphPad Prism. In particular, the average overall activity over 1 h and 40 min and the ON and OFF peaks (activity recorded during the first 5 sec following the light change) were quantified for each treatment group. The activity of individual larvae was measured in milliseconds per second (ms/s).

### RT-PCR

Eye dissection was carried out after 4 and 24 hours of 20 µg/mL CSE exposure. Before the dissection, 12 drug-treated larvae were washed three times with fresh embryo medium and stored in RNAlater (Merck) overnight at 4°C. Dissected eyes were homogenized through a 26-gauge needle/syringe and total RNA was extracted using the mirVana miRNA Isolation Kit (Thermo Fisher Scientific) according to the manufacturer’s instructions. RNA samples were precipitated using 100% ethanol and 3 M sodium acetate solution overnight at −20 °C, then washed with 80% ethanol. Pellets were dissolved in nuclease-free water. The concentration was measured using a Denovix DS-11 spectrophotometer. cDNA was synthesized using a PrimeScript RT reagent Kit (Perfect Real Time) (TaKaRa Bio Inc.) according to the manufacturer’s protocol. Quantitative real-time PCRs were carried out using a QuantStudio 7 Flex Real-Time PCR System (Applied Biosystems). Targets were detected using PowerSYBR Green PCR Master Mix (Applied Biosystems) under the following conditions: 50 °C for 2 min, 95 °C for 10 min, then 40 cycles at 95 °C for 15 s and 60 °C for 60 s. Primers used are: *cat*_Fwd: TGAGGCTGGGTCATCAGATA; *cat*_Rev: AAAGACGGAAACAGAAGCGT; *gpx1a*_Fwd: AGGCACAACAGTCAGGGATT; *gpx1a*_Rev: CAGGAACGCAAACAGAGGG; *casp3a*_Fwd: TAGTGTGTGTGTTGCTCAGTC; *casp3a*_Rev: CTCGACAAGCCTGAATAAAG; *nfe2l2a* _Fwd: GAGCGGGAGAAATCACACAGAATG; *nfe2l2a* _Rev: CAGGAGCTGCATGCACTCATCG; *actb1* _Fwd: ACATCCGTAAGGACCTG and *actb1*_Rev: GGTCGTTCGTTTGAATCTC. All reactions were performed in technical triplicates. Relative expression of targets was assessed by the 2-ΔΔCT method using β-actin as the housekeeping gene.

### Hyaloid vessels assay

*Tg(fli1:EGFP)* larvae treated with CSE from 72 to 120 hpf (48 h CSE exposure) were culled using 4% paraformaldehyde (PFA) fixative, gently shaken overnight at 4 °C and washed three times the next day in 1X PBS-0.1% Tween 20 PBST. Whole larvae were screened for overall defects before dissecting the lens using an Olympus SZX16 fluorescent microscope. For analysis, lenses were transferred to 9% methylcellulose on microscope depression slides and reoriented with tweezers and tungsten needles for optimal visualisation of hyaloid vessels as described previously ^31^. The number of main branches radiating from the optic disc area was quantified and their diameter measured by drawing a perpendicular line to the wall of the vessel, right after the point of branching out from the hyaloid artery, using the “arbitrary line” tool in OLYMPUS cellSens Standard software. Three replicates were run on separate days. Each replicate consisted of eight larvae each in the following groups: 0.05% DMSO, 15 µg/mL CSE or 20 µg/mL CSE.

### Histological analysis

For light microscopy, larvae were fixed in glass vials with 2.5% glutaraldehyde, 2% paraformaldehyde (PFA) and 0.1% Sorenson’s phosphate buffer (pH 7.3) and placed at 4°C overnight. Samples were transferred to 1% osmium tetroxide before an ethanol gradient dehydration. Larvae were embedded in agar epoxy resin and sectioned using a glass knife and a Leica EM UC6 microtome. Retinal sections were placed on glass slides and stained with Toluidine blue (Sigma Aldrich, UK) and imaged using a Leica DMLB bright field illumination microscope with a Leica DFC 480 camera. For transmission electron microscopy (TEM), sections from light microscopy were stained with uranyl acetate and lead citrate and imaged with FEI Tecnai 120 transmission electron microscope (Thermo Fisher Scientific, Waltham, MA, United States). Morphological analysis was performed on retinal sections for light microscopy and using arbitrary lines from OLYMPUS cellSens Standard software with SZX2-ILLT Olympus stereo microscope. First, a straight arbitrary line connecting both marginal zones named here front plane was traced. Axial length was measured using a perpendicular line (named optic axis here) to front plane from anterior lens surface to RPE. Retinal layers were all measured at the optic axis. Measurements were done using an Olympus SDF PLAPO 1.6XPF objective and 11.5X magnification.

### Transmission electronic microscopy (TEM) and phagosomes quantification

Samples preparation, imaging for TEM and phagosomes analysis were performed as reported previously ^32^. Briefly, zebrafish samples were prepared for TEM using the same protocol for light microscopy. 80 nm sections were cut on a Leica EM UC6 microtome and mounted on copper grids and post-stained with 2% uranyl acetate and 3% lead citrate. The optic nerve was used as a reference point for sectioning. Imaging was performed on an FEI Tecnai 120 electron microscope. By TEM, phagosomes were manually counted, and the density was calculated as phagosomes per micron of RPE. A minimum of 350 μm of RPE surface was analysed per larva.

### Data analysis

Statistical analysis was completed using GraphPad Prism 7.00 software (GraphPad, San Diego, CA). Statistical analyses of 48 hours-OKR, VA, CS, RGB OKR and Biometry analyses were evaluated using a one-way ANOVA and a Bonferroni’s multiple comparison test, comparing CSE-treated larvae to vehicle controls. 24 hours-OKR and phagosomes quantification were analysed by unpaired t-test. Visualmotor response assay data was analysed by unpaired t-test and a non-parametric Mann-Whitney test. RT-PCR analysis was evaluated with one-way ANOVA and a Dunnett’s multiple comparison test. Hyaloid vessels statistical analysis was carried out using Kruskal-Wallis test followed by Dunn’s multiple comparisons. Significance levels were set at p<0.05.

## Results

### Systemic cigarette smoke extract attenuates zebrafish visual function

**Figure 1.**
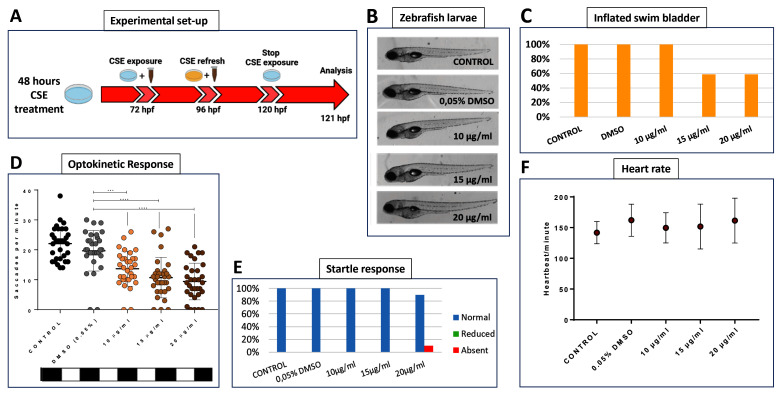
Cigarette Smoke Extract Impairs Visual Behaviour. **A**. Schematic illustrating the timeline and experimental design of 48 hours cigarette smoke extract (CSE) treatment of zebrafish larvae and physiological analysis. **B**. Gross morphology of 5 dpf larvae treated with CSE showing no macroscopic abnormalities except for the incomplete inflation of swim bladder. **C**. The percentage of larvae with inflated swim bladders is only reduced at 15 or 20 µg/mL CSE, n=24 larvae per group, experiment repeated 3 times. **D**. Standard optokinetic response assay (0.02 cpd) at 5 dpf shows visual behaviour is significantly affected at 10, 15 and 20 µg/mL CSE; n=32 larvae per group, experiment repeated 4 times. Data were analysed by one-way ANOVA and Bonferroni´s multiple comparison test where **=p<0.01, ***=p<0.001 and ****=p<0.0001. Error bars indicate standard deviation (SD). **E**. Normal swimming response when caudal fin was touched at 5 dpf. N=16 larvae per group, experiment repeated 2 times. **F**. Heartbeat per minute average reveals no significant effects of CSE on cardiac rhythm at 5 dpf. N=8 larvae per group, experiment repeated 2 times.

To identify doses of CSE that selectively induce effects on vision, a dose-response analysis was undertaken exposing 72 hpf wild-type zebrafish larvae to CSE for 48 hours, before examining gross macroscopical morphology, visual behaviour, motility and cardiac function ( Figure 1). CSE doses higher than 20 µg/mL were toxic (Data of zebrafish larvae treated with lethal doses of CSE are not included due to the severe state of decomposition, which rendered them unsuitable for presentation and analysis) with larvae displaying abnormal development, extensive oedema or lack of mobility. More specifically, concentrations of 40 and 50 µg/mL CSE were lethal, and 30 µg/mL CSE invoked developmental delay (as above, data not shown). Based on macroscopic assessment, larvae treated with 10, 15 or 20 µg/mL CSE displayed grossly normal morphology ( Figure 1B). However, 41% of larvae treated with 15 or 20 µg/mL CSE did not display properly inflated swim bladders ( Figure 1B–C). Swim bladders are inflated by zebrafish swimming-up to the water-air interface and an uninflated swim bladders are often observed in zebrafish with impaired vision ^33, 34^.

The OKR is an established visual behaviour assay widely used for assessment of vision in zebrafish ^16, 35^. Larvae treated with 10, 15 or 20 µg/mL were assessed using a 0.02 cpd drum, with 100% contrast, considered the *standard optokinetic response* ( Figure 1D). A dose-dependent reduction in visual behaviour was observed with CSE. Larvae treated with 10, 15 or 20 µg/mL CSE showed an average of 13.7, 10.7 or 9.4 saccades per minute, respectively, which were significantly lower compared with the 0.05% DMSO vehicle control group (p=0.0093, p=0.0004 and p<0.0001, respectively) that exhibited 19.7 saccades per minute. ( Figure 1D).

To assess the selectivity of the CSE effect on visual behaviour, other more general, systemic readouts of physiology were analysed. The touch or startle response is a locomotor test assessing general motility ^16, 36^. Larvae treated with 10 or 15 µg/mL CSE showed a normal touch response, and only one of 16 larvae treated with 20 µg/mL CSE showed an absent touch response ( Figure 1E). Cardiac physiology assessed by heart rate was not altered in any of the 10, 15 or 20 µg/mL CSE-treated groups (149.63, 151.75 or 161.38 bpm, respectively) when compared with the 0.05% DMSO and untreated control groups (141.75 or 162 bpm, respectively) ( Figure 1F). In conclusion, specific concentrations of CSE appear to induce a selective visual behaviour deficit in zebrafish larvae. 

### Cigarette smoke extract attenuates visual acuity and contrast sensitivity

**Figure 2.**
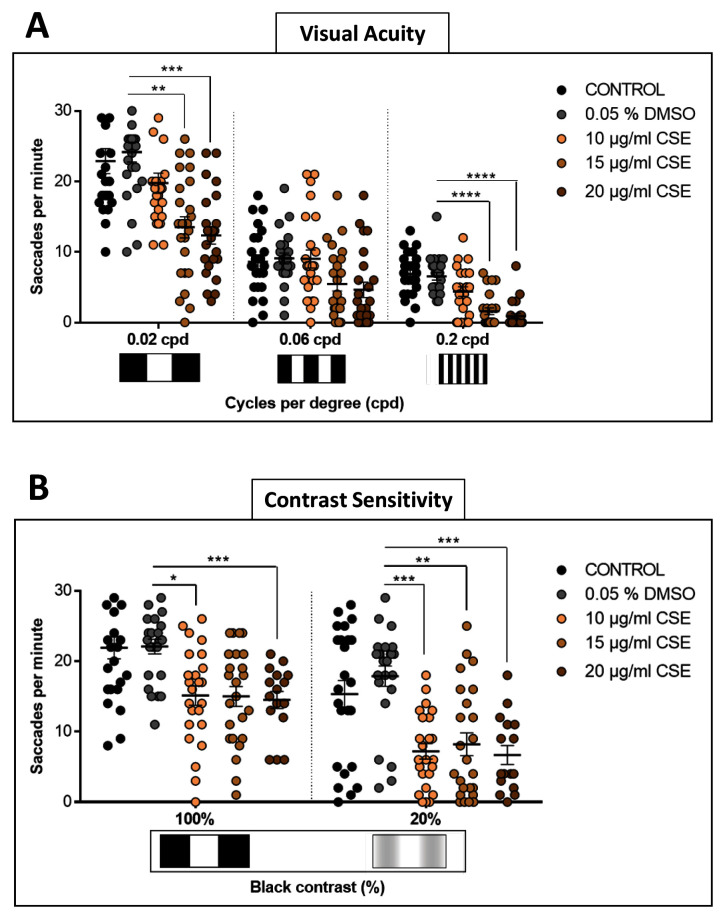
Visual Acuity and Contrast Sensitivity are affected by Cigarette Smoke Extract Treatment. **A**. Larvae treated with 15 or 20 µg/mL CSE have a decreased visual acuity response to 0.2 cpd, 100% contrast drum, compared to DMSO treated larvae. A Levene's test was performed and the variance across 0.06 cpd groups is not statistically significantly different (p=0.77). **B**. For contrast sensitivity, all CSE concentrations tested result in a significantly reduced number of saccadic eye responses with the 20% black contrast drum, compared to DMSO controls. Data were analysed by repeated measures one-way ANOVA and Bonferroni’s multiple comparison test, where *=p>0.01, **=p<0.01, ***=p<0.001 and ****=p<0.0001. Error bars indicate standard deviation (SD). Midline of error bars represents the group average. *Protocol I* used, three replicates of eight larvae, n=24 measurements per pattern per group.

To assess effects of CSE on visual behaviour in more depth, visual acuity (VA) and contrast sensitivity (CS) were assessed ( Figure 2). To examine VA *,* each larva was assessed consecutively with a 0.02, then a 0.06, and finally a 0.2 cpd drum, all with 100% contrast ^19^. The responses for the 0.02 cpd drum show CSE-treated larvae with reduced saccadic eye movements. Responses of 13.5 and 12.3 saccades per minute, respectively with the 15 and 20 µg/mL CSE-treated larvae were significantly lower (p=0.0015 and p=0.0001, respectively) than the 0.05% DMSO control group which evoked 24.2 saccades per minute ( Figure 2A). Surprisingly, although the average OKR to the mid-spatial frequency drum of 0.06 cpd, was lower for the 15 and 20 µg/mL CSE-treated larvae than the DMSO vehicle control group, this was not statistically significant ( Figure 2A). However, the higher doses, 15 or 20 µg/mL CSE, also significantly impaired (p<0.0001) the ability of zebrafish larvae to respond to the highest spatial frequency tested with the 0.2 cpd 3D-printed drum ( Figure 2A), lowering the number of saccades per minute to 1.6 and 0.9, respectively, *versus* the DMSO vehicle control group which responded with 6.5 saccades per minute.

CS was tested comparing 0.02 cpd drums but with 100% or 20% contrast ^19^. With the 20% black-white contrast drum, all zebrafish groups treated with CSE displayed significantly decreased visual responses ( Figure 2B). CSE-treated groups with 10, 15 and 20 µg/mL showed 7.2, 8.2 and 7.2 saccades per minute, respectively, which were significantly lower (p=0.0006, p=0.0043 and p=0.0002 respectively) than the 0.05% DMSO vehicle control-treated larvae which respond with 17.9 saccades per minute. In summary, these bespoke visual behaviour assays showed that CSE impacts visual acuity and contrast sensitivity in zebrafish.

### Cigarette smoke extract-treated larvae show diminished saccadic responses to coloured drums

**Figure 3.**
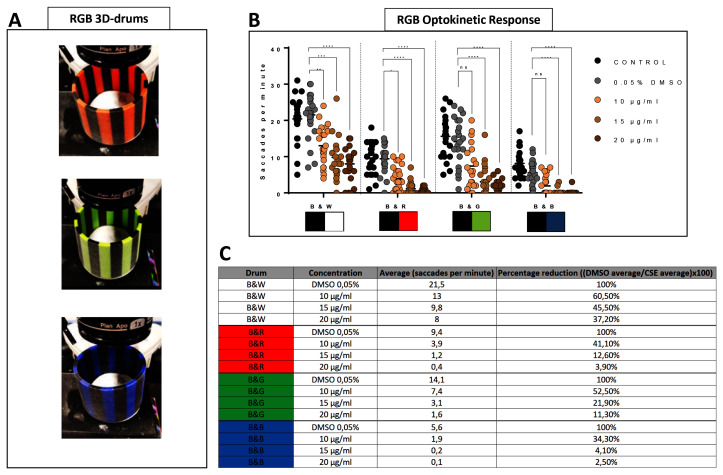
Cigarette Smoke Extract decreases the ability to discriminate colour patterns. **A**. Larvae treated with 10, 15 and 20 µg/mL have a poorer optokinetic response to customised RGB 0.02 cpd drums. **B**. Average of number of saccades and reduction between 0.05% DMSO control group and CSE-treated larvae. **C**. Table showing OKR response from CSE-treated larvae to Red-Black, Green-Black and Blue-Black G response is lower than standard OKR (black-white stripes). Data were analysed by repeated measures one-way ANOVA and Bonferroni’s multiple comparison test where ****=p<0.0001 and **=p<0.01. Error bars indicate standard deviation. Midline of error bars represents the group average. *Protocol I* ( 19 used, three replicates of eight larvae, n=24 measurements per pattern per group.

To investigate if CSE treatment selectively affects optokinetic responses to coloured patterns, visual behaviour responses to drums with coloured stripes contrasted with alternating black stripes were quantified ( Figure 3A). Thus, larvae treated with 10, 15 or 20 µg/mL CSE were confronted with rotating black-white ( *standard*), then black-red, black-green or black-blue 0.02 3D-printed drums ( Figure 3). As shown earlier, larvae treated with CSE display reduced saccades with the standard OKR black-white 0.02 drum ( Figure 1D, Figure 3B). When testing the black-red drum, 10, 15 or 20 µg/mL CSE-treated larvae generated significantly impaired visual responses; reduced by 82% (3.9 saccades per minute, p=0.0126), 94.5% (1.2 saccades per minute, p<0.0001) and 98.3% (0.4 saccades per minute, p<0.0001), respectively compared to the 0.05% DMSO vehicle control larvae ( Figure 3B–C). With the black-green drum, 15 µg/mL CSE significantly reduced the number of saccades by 85.6% (3.1 saccades per minute, p<0.0001) and 20 µg/mL CSE by 92.6% (1.6 saccades per minute, p<0.0001). With the black-blue drum, 15 and 20 µg/mL CSE-treated larvae significantly reduced their saccades per minute by 98.9% and 99.4%, respectively (p<0.0001 and p<0.0001) ( Figure 3B–C). Overall, the biggest absolute reduction in visual behaviour was observed with the black-white drum wherein 20 µg/mL CSE displayed an average reduction of 13.5 saccades per minute, followed by black-green, black-blue and black-red with saccade per minute reductions of 12.5, 9 and 5.5, respectively ( Figure 3C). Notably, the rank order of the largest percentage reduction in OKR was with the black-blue, black-red, black-green and black-white drum when exposed to 20 µg/mL CSE ( Figure 3C).

### Short cigarette smoke extract exposure induces abnormal visual behaviour

**Figure 4.**
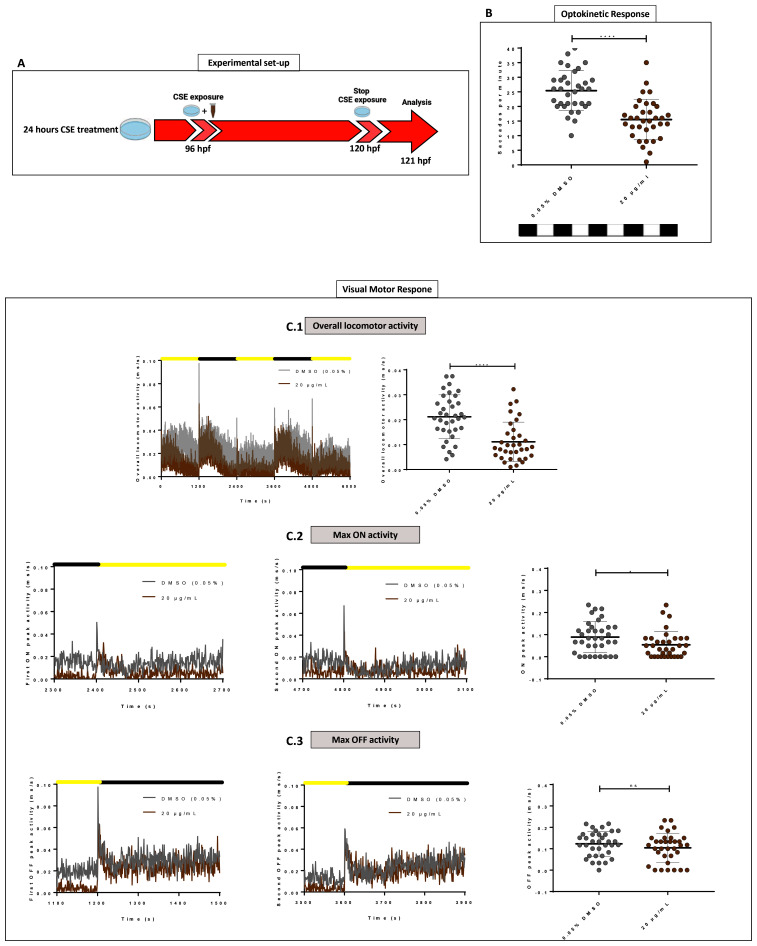
Standard Optokinetic Response and Visual Motor Response are affected when CSE is applied for 24 hours. **A**. Schematic illustrating the timeline and experimental design of 24 hours cigarette smoke extract (CSE) treatment of zebrafish larvae. **B**. Standard optokinetic response (0.02 cpd, 100% contrast) at 121 hpf after 24 hours CSE-treatment. Larvae treated with 20 µg/mL show a lower optokinetic response than 0.05% DMSO vehicle control group. Statistical analysis carried out by student t-test between groups where ****=p<0.0001. Midline of error bars represents the group average. Three replicates, n=36 measurements per treatment group. **C**. Visualmotor response traces showing overall locomotor activity ( **C.1**) and max ON ( **C.2**) and max OFF ( **C.3**) peak activities revealed 20 µg/mL CSE-treated larvae display less overall locomotor movements ( **C.1**) but still react to light changes ( **C2–C3**). Statistical analysis was carried out by unpaired t-test and a non-parametric Mann-Whitney test where *=p>0.01 and ns=no significant. Error bars indicate standard deviation and midline represents the group mean. 3 replicates of twelve larvae were used, n=36 measurements per group.

To analyse shorter-term effects of CSE, *wild-type* zebrafish larvae were exposed at 96 hpf to CSE for only 24 hours and the standard optokinetic response (0.02 cpd, 100% contrast) performed at 120 hpf ( Figure 4A–B). A 20 µg/mL CSE concentration was chosen as it produced the most adverse effects on visual behaviour in the earlier assays with 48 hours CSE treatment. A 24-hour exposure to CSE resulted in larvae exhibiting a significantly lower number of eye movements (15.53 saccades per minute, p<0.0001) compared with vehicle controls (25.41 saccades per minute). Following the same exposure time ( Figure 4A), the VMR was assessed in larvae treated with 20 µg/mL CSE-treated or 0.05% DMSO vehicle control ( Figure 4C). The VMR is a visual behaviour assay that analyses how zebrafish acutely respond to lights turned ON and OFF ^16, 37, 38^. Here, we analysed overall locomotor activity and peak locomotor activity when lights go ON and OFF ( Figure 4C). In the overall activity, 20 µg/mL CSE results in significantly lower (52.5% reduced, p<0.0001) larval activity than the 0.05% DMSO vehicle group. ( Figure 4C.1). CSE treated larvae showed 0.01±0.008 milliseconds per second (ms/s) average activity, versus the 0.05% DMSO vehicle group, which showed 0.021±0.009 ms/s average activity. In relation to changes in locomotor activity when lights were switched ON/OFF, although CSE-treated larvae exhibited markedly reduced activity before either change in light ( Figure 4C.2–3), they could sense the lighting status and did increase their activity, albeit not to the same level as the DMSO controls ( Figure 4C.2–3). In relation to peak activity at the light changes, there was no significant change in the average maximum peak OFF activity between 20 µg/mL CSE-treated larvae (0.1±0.07 ms/s) compared to vehicle controls (0.12±0.058 ms/s) ( Figure 4C.3). However, CSE treatment resulted in a significant reduction in the average maximum peak ON activity (0.05±0.06 ms/s activity, p=0.0342) compared to 0.05% DMSO controls (0.09±0.07 ms/s) ( Figure 4C.2).

### Four-hour and 24-hour CSE treatment alters gpx1a expression in larval zebrafish eyes

**Figure 5.**
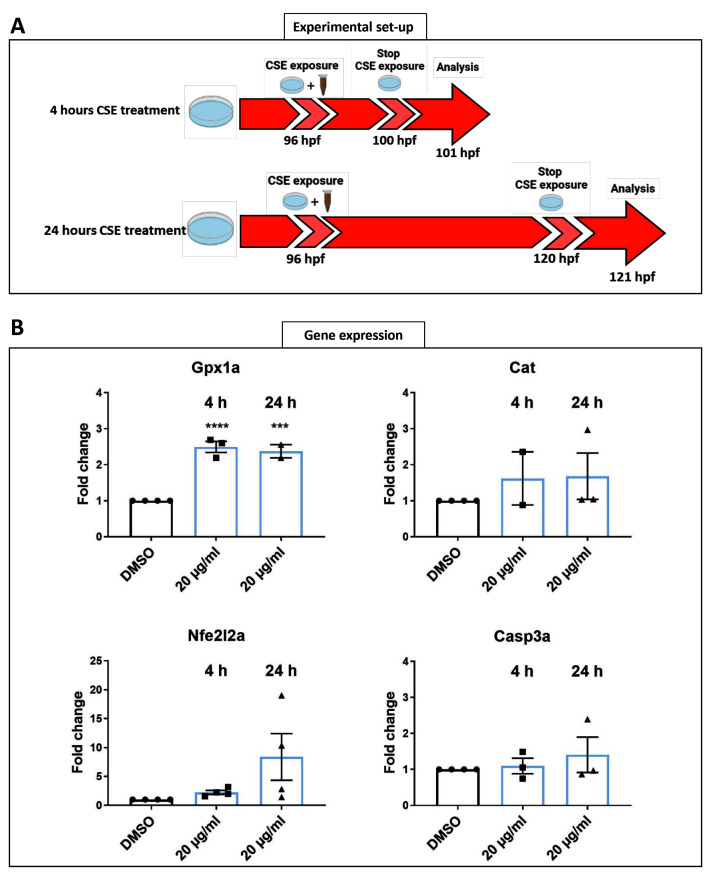
Shorter CSE treatment increases gpx1a expression. **A**. Schematic representation of the timeline and experimental design of 4- and 24-hours cigarette smoke extract (CSE) treatment of zebrafish larvae. **B**. Gene expression profiles revealed a significant higher expression of gpx1a at 4- and 24- hours 20 µg/mL CSE treatment in zebrafish larvae eyes. Data was analysed by one-way ANOVA and a Dunnett’s multiple comparison test where ****=p<0.0001 and ***=p<0.001. 3 replicates of twelve larvae were carried out, n=72 larval eyes per group.

Smoking and cigarette smoke cause oxidative damage to the eye and increase the risk of AMD and cataracts by increasing oxidative stress ^6, 39– 41^). Here, we analysed the following primary antioxidant genes: catalase ( *cat*) and glutathione peroxidase 1 ( *gpx1a*), as well as nuclear factor erythroid 2–related factor 2 ( *nfe2l2a*), related with cellular defence against oxidative damage, and caspase 3 ( *casp3a*), the major caspase activated by apoptosis ( Figure 5). A total of 24 enucleated larval eyes, treated for four or 24 hours ( Figure 5A) with CSE, were used for RT-PCR reactions. Significant changes in *cat, nfe2l2a and casp3a* were not observed after four or 24 hours CSE treatment ( Figure 5B). *gpx1a* expression increased significantly after either four (p=0.0001) or 24 hours (p=0.0003) of CSE treatment compared with 0.05% DMSO vehicle control larvae ( Figure 5B). This suggests that CSE modulates oxidative stress in the eye.

### Hyaloid vessel diameter increases after 48-hours CSE exposure

**Figure 6.**
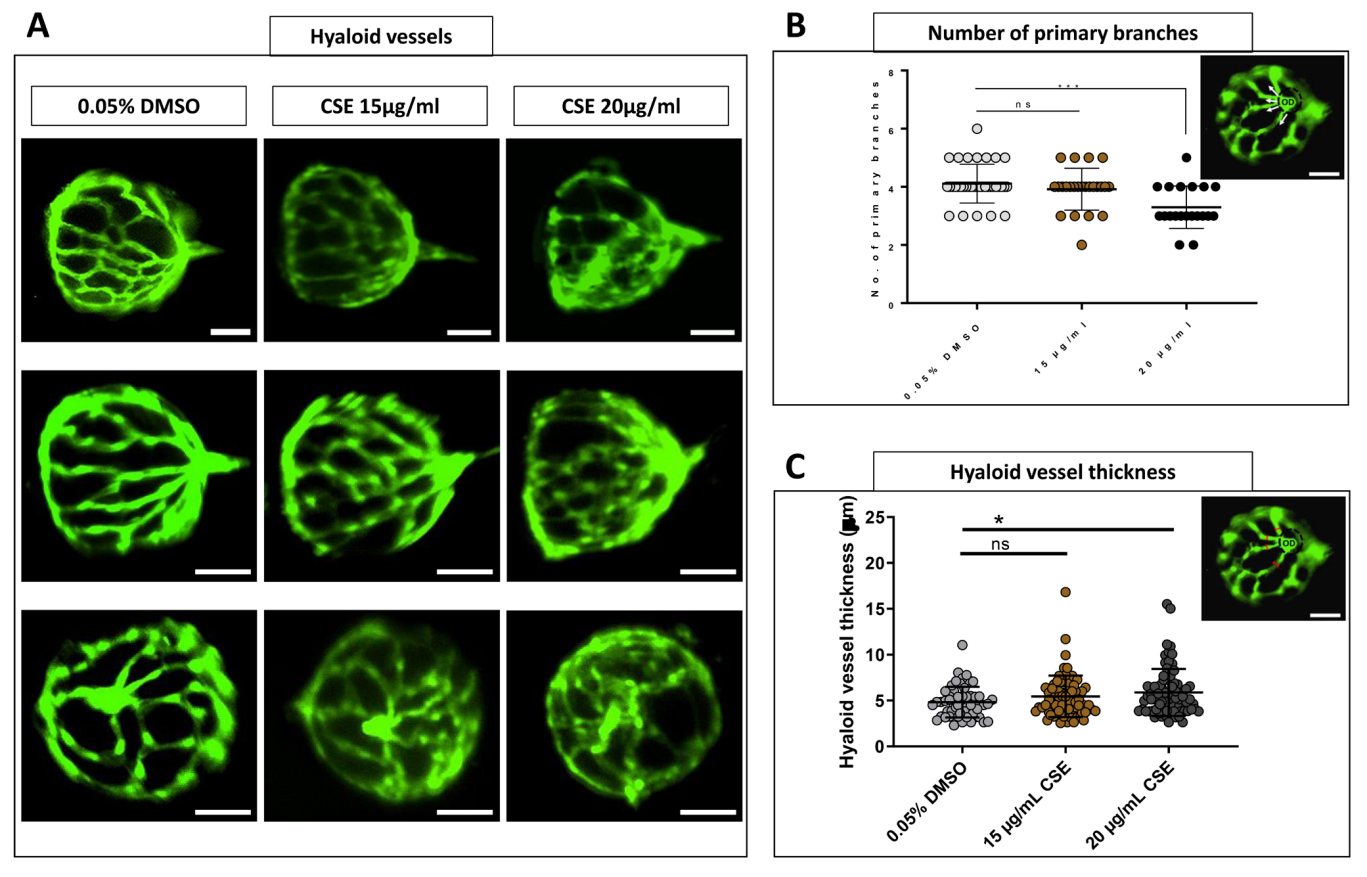
Hyaloid vessels are dilated with 48 hours Cigarette Smoke Extract Systemic Treatment. **A**. Fluorescent images of hyaloid vasculature on 0.05% DMSO vehicle control group and 15 and 20 µg/mL CSE treated dissected lenses. Dilated and non-defined vessels are evident in 15 and 20 µg/mL CSE-treated lenses. Scale bar= 50 µm. **B**. 20 µg/mL CSE-treated zebrafish larvae develop a smaller number of primary branches. Data was analysed by Brown-Forsythe, Welch ANOVA tests and Dunnett’s T3 multiple comparisons test where *p<0.05, ***=p<0.001 and ns=no significant. C. Hyaloid vessel thickness plot graph revealed an abnormal increase of hyaloid vessel thickness in 15 and 20 µg/mL CSE-treated zebrafish larvae. White arrows indicate primary hyaloid branches. Data was analysed by Kruskal-Wallis test and Dunn’s multiple comparisons tests where *=p<0.05 and ns=no significant. Delimiter red lines define where thickness of hyaloid vessels was measured. OD: Optic disk. Five biological replicates, minimum 55 measurements per group.

To investigate the effect of CSE on developing intraocular vessels in zebrafish, *Tg(fli1:EGFP)* larvae were treated for 48 hours with 15 or 20 µg/mL CSE, prior to morphological analysis of the hyaloid vasculature (HV). The HV develops in zebrafish from 24 to 72 hpf and consists of four to five main branches radiating from the hyaloid artery which enters through the optic disc at the back of the lens. These primary vessels branch out multiple times and eventually drain to the inner optic circle at the anterior surface of the lens, which remains avascular. By 5 dpf, hyaloid vasculature constitutes an intricately branched network of intraocular vessels that nourish the developing lens and inner retina ^31, 42, 43^. The following measurements were conducted, acknowledging that the compromised visibility of some of the vessels was attributed to structural damage and dilation induced by CSE Systemic treatment ( Figure 6A). The number of primary hyaloid branches radiating from the optic disc was not altered in larvae treated with 15 µg/mL CSE, whereas 20 µg/mL CSE decreased this number modestly (p=0.002) compared to DMSO controls ( Figure 6B). Direct observation under the microscope suggested the hyaloid vessels in both 15 and 20 µg/mL CSE-treated groups, appeared thicker than the DMSO vehicle controls ( Figure 6A). Thus, hyaloid vessels were measured at the primary branch level, between the first bifurcation at the optic disc and the second bifurcation ( Figure 6A). The diameter of the vessels increased, although not significantly, from 4.833 µm in 0.05% DMSO vehicle control group to 5.454 µm in 15 µg/mL CSE-treated larvae. However, the diameter of the vessels in 20 µg/mL CSE-treated larvae significantly increased to 5.885 µm (p=0.0333) ( Figure 6C).

### Ocular axial length and lens thickness increase with CSE exposure

**Figure 7.**
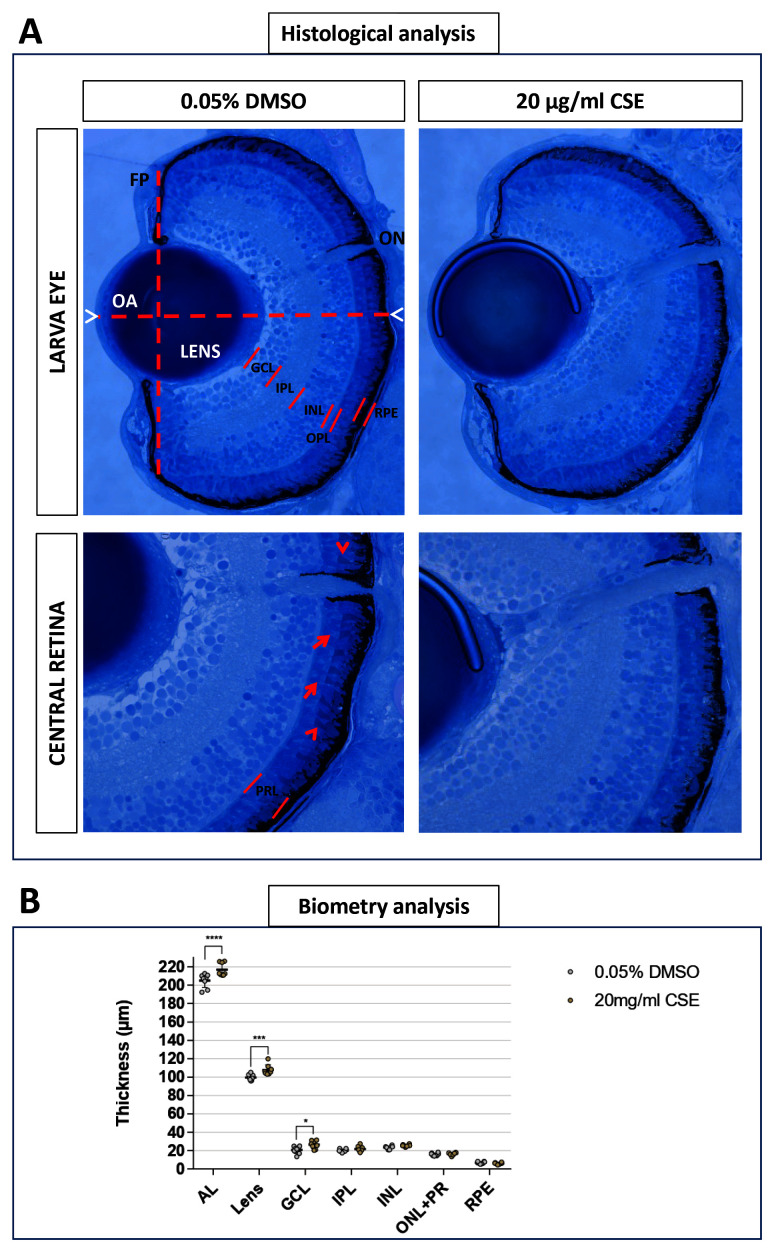
Axial Length, Lens and Ganglion Cell Layer appear increased with 48 hours Cigarette Smoke Extract Systemic Treatment. **A**. 20 µg/mL CSE treated larvae do not display observable defects in eye morphology or retinal arrangement. Representative light microscopy images from 0.05% DMSO control and 20 µg/mL CSE-treated larvae. Front plane (FP) and Optic Axis (OA) traced for morphological analysis are defined by dashed lines. Axial length (AL) is delimited by white arrowheads. Retinal layers are defined by red lines. PRL is formed by both photoreceptors inner segments (red arrow) and outer segments (red arrowhead). **B**. Quantitative biometry of retinal layers using Olympus cellSens standard software, showed enlarged AL and lens in 20 µg/mL CSE-treated larvae, n=8 larvae analysed. Data was analysed by one-way ANOVA and a Bonferroni’s multiple comparison test where ****=p<0.0001 and **=p<0.01. GCL: ganglion cell layer; IPL: inner plexiform layer; INL: inner nuclear layer; OPL: outer plexiform layer; PRL: photoreceptors layer; RPE: retinal pigment epithelium; ON: optic nerve.

Eye histology was imaged to see whether eye structures were affected in 121 hpf larvae treated with 0.05% DMSO or 20 µg/mL CSE for 48 hours ( Figure 7). Retinal layers were well-defined and retinal cell morphology appear not be affected by 20 µg/mL CSE ( Figure 7A). Interestingly, biometry analysis highlighted a statistically significant axial length elongation in 20 µg/mL CSE-treated larvae (216.9 µm, p<0.0001) compared to 0.05% DMSO vehicle control (205.1 µm) and a lens thickness increase, to 107.9 µm (p=0.0044) in CSE-treated larvae compared to 99.65 µm in the 0.05% DMSO vehicle control group ( Figure 7B). No differences were found in the thickness of the ganglion cell, inner plexiform, inner nuclear, outer nuclear or retinal pigmented epithelium layers ( Figure 7B).

### CSE increases the number of retinal pigment epithelium phagosomes

**Figure 8.**
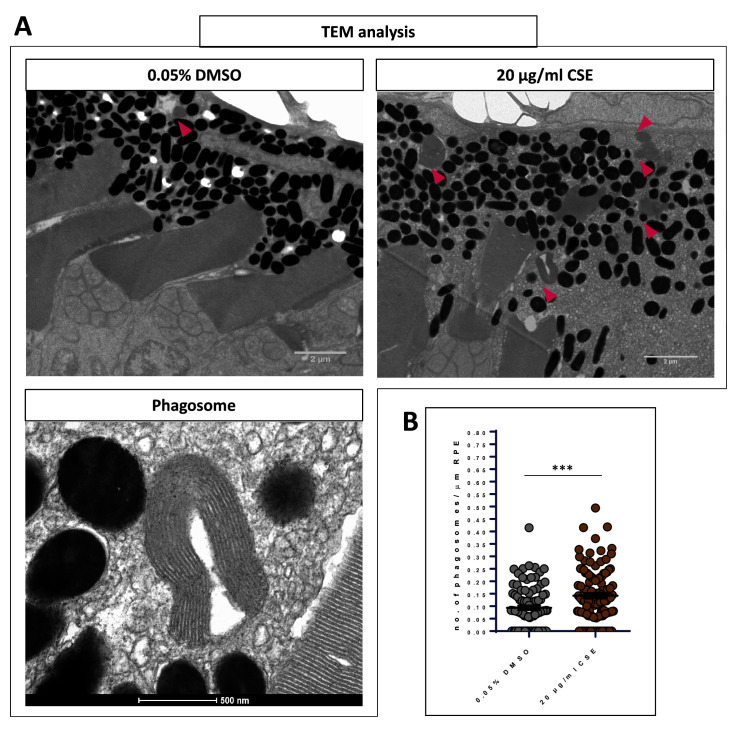
Exposure to cigarette smoke extract causes increased levels of RPE phagosomes. **A**. Representative TEM images of *wild-type* retinae at 121 hpf showing the OS tip and RPE of larvae treated with 0.05% DMSO and 20 µg/mL CSE of CSE. Phagosomes are indicated (pink arrows). Scale bar; 2 µm. Magnified TEM image of a phagosome. Scale bar 500 nm. **B**. Scatter plots of phagosome/µm of RPE in 20 µg/mL CSE-treated and vehicle control (0.05% DMSO) retinae. Larvae exposed to CSE for 48 hours show a 50% increase in the number of RPE phagosomes containing engulfed OS material in comparison to the vehicle control controls. Statistical analysis carried out by student t-test between groups where ***=p<0.001. RPE of >3000 μm was measured per larva. N=3,4 larvae (6-8 retinae) per group.

Previous studies in mammals showed CSE negatively affects the RPE ^26, 27^. As the RPE is one of the first retinal structures affected in AMD patients ^41^ and given the relation between AMD and smoking ^3, 13, 14^, we imaged the outer retina of 48 h CSE-exposed zebrafish by TEM. Larvae were fixed between 120-121 hpf, which coincides with the dawn peak of OSP phagocytosis in older zebrafish ^32, 44^ ( Figure 8). Macroscopically, the outer retinae of 20 µg/mL CSE-treated larvae showed a slight disarray of the photoreceptor layer and RPE, and an elevated number of RPE phagosomes ( Figure 8A). Interestingly, upon quantification, larvae exposed to CSE had significantly higher numbers of RPE phagosomes compared to vehicle controls (p=0.0002). 20 µg/mL CSE-treated larvae had an average of 0.1425 ± 0.009287 phagosomes/um RPE (n=4), a value ~50% higher than larvae treated as the vehicle control (0.093 ± 0.009 phagosomes/um RPE, n=3) ( Figure 8B). This suggest that exposing zebrafish larvae to CSE causes dysregulation of OSP.

## Discussion

Cigarette smoke is well characterised as a serious health hazard ^1, 2, 45^. Previous *in vitro* and *in vivo* studies (with human cell lines, mice and zebrafish) investigated the impact of cigarette smoke on the cardiorespiratory system, on vasculature ^24^, and lungs ^46, 47^, as well as its relation with oxidative stress ^48, 49^. However, effects of cigarette smoke on the eye have only been researched in mice ^25– 27, 50^. To our knowledge, this is the first study investigating adverse events of cigarette smoke on the developed zebrafish visual system. Zebrafish are an advantageous alternative vertebrate animal model to analyse cigarette smoke impact on the ocular system, with a high degree of structural and functional conservation between the human and zebrafish retina ^18, 20^.

### CSE treatment of zebrafish larvae impairs vision

In our experimental design, we treated wild-type larvae at 72 hpf to rule out general developmental effects. At this time point, the zebrafish visual system is considered “mature”, with all retinal layers formed and the hyaloid vasculature network established ^18, 31, 51^. The observed maximum tolerated dose of 20 µg/mL matches with response profiles reported in a previous teratogenic study induced by treating 6- to 72-hpf zebrafish larvae with CSE ^21^. The teratogenic study by Ellis *et al.* ^21^ showed a decreased behavioural response to light-dark changes, which the authors attributed to alterations in neuromuscular function or neurotoxicity.

To our knowledge, this is the first study reporting an abnormal visual behaviour in zebrafish treated with CSE. In our model, we observed uninflated swim bladders with 15 or 20 µg/mL CSE. Previous studies report that visually impaired zebrafish often fail to inflate their swim bladder ^34, 52^, conceivably due to difficulty to swim to surface to inhale air. To analyse if CSE affected larvae vision, we conducted variants of the optokinetic response assay (OKR), an efficient and well-established visual behaviour test which can be accurately measured in zebrafish from three days post-fertilization ^35^. CSE significantly impaired zebrafish visual behaviour, visual acuity and contrast sensitivity. Responses to RGB-black coloured drums were also impaired by CSE treatment.

It was important to assess if CSE in our model was inducing broad systemic affects. Previous studies with nicotine or smoke show malformation of the zebrafish heart when treating with CSE at earlier embryonic stages ^22, 24^ and a 50% reduction of heart rate ^21, 22^. In our model, heart rate was not affected in CSE-treated larvae with similar values to the 0.05% DMSO vehicle controls and the untreated larvae. In summary, the reduced zebrafish optokinetic responses ( *e.g.* VA, CS and RGB OKR drum assays) observed appear due to a selective defect in the visual system induced by CSE treatment at 3 dpf. The impaired optokinetic visual behaviour was corroborated by altered visualmotor responses, in which larvae display bursts of movement upon light changes ^16^. Interestingly, previous locomotor activity assays on CSE-treated zebrafish ^23^ found that 24 hpf embryos treated for 20 hours showed hyperactivity to 10 min light changes cycles ^23^. In contrast, Ellis *et al.* ^21^ reported that responses to short 5 min, lights ON and OFF cycles were not impacted by 20 µg/mL CSE (although higher concentrations did at) in 5 dpf larvae treated for 48 hours ^21^. Here, when using longer light cycles (20 min intervals), providing zebrafish more time to adapt between acute light changes, we found overall locomotor activity was lower in zebrafish larvae exposed to 20 µg/mL CSE. For light to dark changes (peak OFF activity), the zebrafish showed a CSE concentration-dependent reduction in locomotor responses ^21^.

### CSE increases anti-oxidant gene expression in zebrafish eyes

Having identified CSE-induced visual behaviour defects that were not linked to widespread systemic effects, we sought to understand the molecular, cellular, and physiological changes in the eye linked to impaired vision. Smoking causes oxidative damage due to free radicals ^6, 7, 39^ which imbalance the antioxidant system and generate reactive oxygen species (ROS) that underlies the pathogenesis of several human eye diseases ^53, 54^. Investigations into the pathophysiology of cigarette smoke-induced AMD revealed that cigarette smoke exposure can generate oxidative stress by producing free radicals, subsequently leading to a depletion of the antioxidant system ^39, 55, 56^. A previous study treating zebrafish larvae from 2 hpf and analysing the activity of ROS biomarkers at 96 hpf found evidence of increased oxidative stress, however, *gpx1a* and *cat* activities were not significantly altered in CSE-treated larvae <96 hpf ^23^. Here, we analysed antioxidant gene expression changes in zebrafish larval eyes treated with CSE from 96 to 101 and to 121 hpf. *Cat* gene expression was unchanged in agreement with the Massarsky *et al.* study ^23^. However, in 101 and 121 hpf CSE-exposed zebrafish, a significantly higher expression of *gpx1a* was observed indicating CSE exposure induces oxidative stress in zebrafish larval eyes. Oxidative damage is a hallmark of AMD development ^27, 53– 55^. Moreover, several studies ^39, 48, 49^ report cigarette smoke-induced oxidative stress in animal models to show similar changes in the eye as AMD patients. Thus, CSE-exposed zebrafish provides an alternative model to study CSE gene expression changes in the eye and associated ocular pathologies.

### CSE alters zebrafish retinal histology

Interestingly, axial length was significantly increased in zebrafish larvae exposed to the highest dose of CSE (20 µg/mL). Myopia is a refractive error where the eye has excessive axial length ^57^. Myopia is a multifactorial condition so environmental factors are involved in its development ^57^. The relationship of CSE exposure and smoking with myopia has been investigated, however, this association remains unclear ^57– 59^. Previous clinical studies found that children of smoking parents had a lower axial length compared with those of non-smokers ^57, 58^. However, in contrast, in chicks administered with nicotinic antagonist drugs ^59^, researchers found that nicotinic receptors are involved in excessive ocular growth. This contrasts with clinical results in humans; nevertheless, nicotine only represents a small percentage in CSE composition ^60^.

Analysis of the retinal layers revealed that the lens had the most significant increase in thickness upon CSE exposure. Smokers have an increased risk of developing nuclear cataracts ^40, 61^, an opacification in the middle of the lens, which is the leading cause of blindness worldwide ^61^. Oxidative stress mechanisms and accumulation of cadmium, iron and other metals presents in the CSE play an important role in cataractogenesis ^61– 63^. For instance, the exposure of rats to cigarette smoke for two hours a day over 60 days, lead to an increase in the number, size and layering of cells in the lens epithelium ^62^. Lens thickness is not usually a clinical practice to diagnose cataracts but it is known the lens becomes thicker in the presence of nuclear cataracts ^64^. We speculate that the CSE zebrafish model may be experiencing an onset of similar biological effects in the lens, however, further histological and biomolecular analysis are needed.

### Systemic CSE treatment induces enlarged hyaloid vessels

The zebrafish HV surrounds the larval lens and becomes attached to the inner surface of the retina in the adult (after 28 dpf) ^31^. The HV support development of the lens and retina, and share many features with human retinal vasculature, making them a valuable model of human disease ^31, 43, 65^. In 5-dpf larvae, exposure to CSE for 48 hrs, HV appear dilated and display a significantly enlarged thickness ( Figure 6C). We also observed a reduction in the number of main HV branches ( Figure 6B). As these were probably completely formed when CSE was administered at 72 hpf, this is likely a toxic effect rather than an antiangiogenic effect ^31^. In mammals, systemic vascular tone dysfunction and vascular inflammation induced by CSE exposure (even short term) is described in the literature, and also associated with second-hand smoking ^66, 67^. Retinal vascular calibre is a well-established biomarker of cardiovascular disease (including diabetes) ^68, 69^, and in ophthalmology, age-related macular degeneration (AMD) is associated with arteriolar and venular dilation in the retina ^70^. Many studies have researched the effects of cigarette smoking (including second-hand) on vessels in the retina and the choroid. Enlarged retinal calibre is extensively related to cigarette smoking in the literature ^71, 72^, although it has also been linked to narrower retinal arterioles and venules in certain cases ^72– 74^. The unanimous consensus in the literature is that cigarette smoke induces alterations in the microvascular calibre of the choroidal and retinal capillary plexus, which have been linked to oxidative stress, inflammation, and increased risk of vascular disease ^71– 74^.

### Photoreceptor outer segment phagosomes increased by CSE

Outer segment phagocytosis (OSP) is a regulated process that maintains photoreceptor health and vision ^32^. Pharmacological modulation can affect the OSP at any stage from recognition, binding or degradation ^75, 76^. Interestingly, exposing zebrafish larvae to CSE causes dysregulation of the OSP process, resulting in higher levels of RPE phagosomes. In general, increased phagosomes and phagolysosomes can indicate two possible mechanisms: either increased engulfment and phagocytosis and/or suppressed degradation. To our knowledge this is the first time CSE exposure has been linked to the OSP process in zebrafish. Cigarette smoke is associated with lysosomal dysfunction, with activity of the known OSP regulator cathepsin D being altered upon CSE exposure in both cell culture and murine models ^56, 77^. The lysosomal activity of cathepsin D is required for the degradation of the removed OS tip, the final stage of OS phagocytosis ^78^. The molecular mechanisms involved in the increased level of RPE phagosomes observed in the CSE treated larvae remain to be elucidated.

## Conclusions

We present here evidence that CSE disrupts visual behaviour, visual acuity and contrast sensitivity in larval zebrafish. Zebrafish eyes showed an increased oxidative stress as well as an abnormal HV. CSE induced axial length elongation, lens thickness increase and a disruption of photoreceptor outer segment phagocytosis. These findings provide zebrafish as a tool to investigate biological changes induced by cigarette smoke in the eye. 

## Data Availability

Zenodo: Systemic Treatment with Cigarette Smoke Extract Affects Zebrafish Visual Behaviour, Intraocular Vasculature Morphology and Outer Segment Phagocytosis, https://doi.org/10.5281/zenodo.10090642 ^79^ This project contains the following underlying data: Figure. 1C-D-F. Cigarette Smoke Extract Impairs Visual Behaviour..xlsx Figure. 1D Figure 1. Cigarette Smoke Extract Impairs Visual Behaviour..pzfx Figure. 1F Figure 1. Cigarette Smoke Extract Impairs Visual Behaviour..pzfx Figure. 4B. Standard Optokinetic Response and Visual Motor Response are affected when CSE is applied for 24 hours..pzfx Figure. 4C. Standard Optokinetic Response and Visual Motor Response are affected when CSE is applied for 24 hours..pzfx Figure. 5. Shorter CSE treatment increases gpx1a expression.pzfx Fig. 6BONLY. Hyaloid vessels are dilated with 48 hours Cigarette Smoke Extract Systemic Treatment..pzfx Figure. 7. Axial Length, Lens and Ganglion Cell Layer appear increased with 48 hours Cigarette Smoke Extract Systemic Treatment..pzfx Figure. 8. Exposure to cigarette smoke extract causes increased levels of RPE phagosomes.pzfx Fig_1C_Cigarette Smoke Extract Impairs Visual Behaviour.csv Fig_1D_Cigarette Smoke Extract Impairs Visual Behaviour.csv Fig_1E_Cigarette Smoke Extract Impairs Visual Behaviour.csv Fig_1F_Cigarette Smoke Extract Impairs Visual Behaviour.csv Fig_2A_Visual Acuity and Contrast Sensitivity are affected by Cigarette Smoke Extract Treatment.csv Fig_2B_Visual Acuity and Contrast Sensitivity are affected by Cigarette Smoke Extract Treatment.csv Fig_3B_Cigarette Smoke Extract decreases the ability to discriminate colour patterns.csv Fig_3C_Cigarette Smoke Extract decreases the ability to discriminate colour patterns.csv Fig_4B_Standard Optokinetic Response and Visual Motor Response are affected when CSE is applied for 24 hours.csv Fig_4C_MAX OFF peak activity_larva.csv Fig_4C_MAX ON peak activity_larva.csv Fig_4C_overall locomotor activity_larva.csv Fig_4C_traces 1 ^st^ OFF peak.csv Fig_4C_traces 1 ^st^ ON peak.csv Fig_4C_traces 2 ^nd^ OFF peak.csv Fig_4C_traces 2 ^nd^ ON peak.csv Fig_4C_traces overall locomotor activity_larva.csv Fig_5B_Caspase3.csv Fig_5B_Catalase.csv Fig_5B_gpx1.csv Fig_5B_Nrf2.csv Fig_6B_Hyaloid vessels are dilated with 48 hours Cigarette Smoke Extract Systemic Treatment.csv Fig_6C_Hyaloid vessels are dilated with 48 hours Cigarette Smoke Extract Systemic Treatment.pzfx Fig_6C_Hyaloid vessels are dilated with 48 hours Cigarette Smoke Extract Systemic Treatment.csv Fig_7_Biometry.csv Fig_8_Exposure to cigarette smoke extract causes increased levels of RPE phagosomes.csv Figure 2. Visual Acuity and Contrast Sensitivity are affected by Cigarette Smoke Extract Treatment..pzf Figure 3. Cigarette Smoke Extract decreases the ability to discriminate colour patterns..pzfx Zenodo: ARRIVE guidelines checklist for “Systemic Treatment with Cigarette Smoke Extract Affects Zebrafish Visual Behaviour, Intraocular Vasculature Morphology and Outer Segment Phagocytosis”, https://doi.org/10.5281/zenodo.7653425 ^80^ Data are available under the terms of the Creative Commons Attribution 4.0 International license (CC-BY 4.0).
