# Supplementary material for: Systemic treatment with cigarette smoke extract affects zebrafish visual behaviour, intraocular vasculature morphology and outer segment phagocytosis
Source: Open Res Eur. 2023 Nov 23;3:48. Originally published 2023 Mar 23. [Version 2] doi: 10.12688/openreseurope.15491.2 (PMC10822043; doi:10.12688/openreseurope.15491.2)
Supplement: Peer review history

# Reviewer report: Reviewer response for version 2

**DOI:** 10.21956/openreseurope.18209.r36406

**Published:** 2024-01-27

**Authors:** Ross Collery

**Affiliations:** 1 Department of Ophthalmology & Visual Sciences, Medical College of Wisconsin Eye Institute, MILWAUKEE, WI, USA

The authors have been most attentive to the feedback from this reviewer, and have implemented several modifications that both their work. Specifically, definitions and explanations of the hyaloid vessel assay were added, and statistical analyses were expanded. Overall, the authors have produced a most useful manuscript detailing how the highly visual zebrafish make a good model system to study the effects of cigarette smoke exposure on retinal cell biology and visual function.

Is the study design appropriate and does the work have academic merit?

Yes

Is the work clearly and accurately presented and does it cite the current literature?

Yes

If applicable, is the statistical analysis and its interpretation appropriate?

Yes

Are all the source data underlying the results available to ensure full reproducibility?

Yes

Are the conclusions drawn adequately supported by the results?

Yes

Are sufficient details of methods and analysis provided to allow replication by others?

Yes

Reviewer Expertise:

Animal models, retinal development, retinal disease, inherited eye disease

I confirm that I have read this submission and believe that I have an appropriate level of expertise to confirm that it is of an acceptable scientific standard.

# Reviewer report: Reviewer response for version 1

**DOI:** 10.21956/openreseurope.16747.r32520

**Published:** 2023-08-01

**Authors:** Balasubramaniam Annamalai

**Affiliations:** 1 Medical University South Carolina, Charleston, South Carolina, USA

The authors investigated the approach of using systemic treatment with cigarette smoke extract affects zebrafish visual behavior, intraocular vasculature morphology, and outer segment phagocytosis

 This is a good attempt to demonstrate how smoking can alter visual functions. The results will be of interest to readers of the vision science community and relevant industry. The study can be further improved in below listed areas.

1. Authors have analyzed the message levels of glutathione peroxidase 1. But it is relevant to make a significant positive correlation between tGSH levels and TBARS (Thiobarbituric acid reactive substances) levels might indicate that high production of ROS resulted in oxidative stress under CSE-treated conditions, 
2. Increased Retinal pigment-driven macrophage activation might correlate with increased VEGF and /or their receptors and this scenario might contribute to their abnormal vasculature in CSE-treated conditions. The manuscript will strengthen further if the authors can address this and make their argument more interesting.  

Is the study design appropriate and does the work have academic merit?

Yes

Is the work clearly and accurately presented and does it cite the current literature?

Yes

If applicable, is the statistical analysis and its interpretation appropriate?

I cannot comment. A qualified statistician is required.

Are all the source data underlying the results available to ensure full reproducibility?

Yes

Are the conclusions drawn adequately supported by the results?

Yes

Are sufficient details of methods and analysis provided to allow replication by others?

Yes

Reviewer Expertise:

Biochemistry and Molecular biology of the eye ( especially Retinal pigmant epitheilum and Retina )

I confirm that I have read this submission and believe that I have an appropriate level of expertise to confirm that it is of an acceptable scientific standard.

# Reviewer report: Reviewer response for version 1

**DOI:** 10.21956/openreseurope.16747.r32521

**Published:** 2023-06-21

**Authors:** Ross Collery

**Affiliations:** 1 Department of Ophthalmology & Visual Sciences, Medical College of Wisconsin Eye Institute, MILWAUKEE, WI, USA

The authors present a very good manuscript detailing the effects of second-hand cigarette smoke on visual function and ocular health using zebrafish as an animal model. This utilization of zebrafish for systemic delivery of CSE is an excellent application of this species that furthers understanding of the toxic effects of tobacco smoke on the developing visual system and on the brain also. These findings will be useful to understand pathologies in human subjects who have grown up in smoky environments.

 The authors detail the findings that occur following treatment of CSE with varying times and concentrations from 3-5 dpf. They see that optokinetic response, visual motor response, and hyaloid vasculature was affected, though importantly, heart rate was not affected, showing both some specificity for targeting of visual function by CSE, and also improving on previous publications that showed negative effects of nicotine/CSE on the zebrafish heart. CSE treatment also alters RPE phagocytosis and/or diminished ability of the RPE to break down phagosomes. Finally, increases in primary antioxidant genes following CSE exposure suggests an increase in oxidative stress in treated eyes.

 It is clear that exposure to the toxic mixture of chemical contained in cigarette smoke has severe negative consequences for the eye and brain. Using an array of physiologically relevant assays, the authors have demonstrated well the specific effects on the vasculature and function of the eye, and of potential post-ocular defects for processing visual input. Experiments are well-controlled, with appropriate statistical analysis and insightful interpretation. No new experiments are necessary or recommended, though this reviewer has some suggestions about how to extract even more useful conclusions. Overall, this study has been designed and executed with a high degree of rigor and the authors are to be complimented.

 There are several minor points that could be clarified, or elaborated on as detailed below. Overall, this publication is very scientifically sound, but small textual edits might increase clarity and understanding.

 Minor points:

- though the means of the 0.06 cpd OKR test were not statistically significant, the variances seem to be. The authors could consider using an F-test to look for differences in variance for 0.06 cpd groups (e.g. Levene Test ( https://www.itl.nist.gov/div898/handbook/eda/section3/eda35a.htm))
- could the fli1:eGFP line identify differences in brain vasculature after CSE exposure that might suggest a mechanism for altered behavior?
- are genes other than cat, gpx1a, nfe2l2a altered? RNAseq analysis might highlight pathways affected, including ones not considered by the authors.
- would zebrafish allowed to recover to juvenile/adult status after CSE continue to show differences in retinal vasculature or behavior? This may be challenging from an AREC standpoint, but could be justified by the importance of understanding the effects of cigarette smoke exposure during early development on human vision and mental health in later life.
- could the authors comment on the degree of contrast between the different colors in the drums e.g. black vs white seems to have higher contrast than black and red, or black and blue. Does this assay truly test chromatic detection limits or contrast sensitivity?
- could the authors speculate on what is causing the increase in axial length of the larval eye after CSE exposure? The lens size appears to be the likely driver, but the sum of all retinal layers may also meet statistical significance criteria. Measuring the perimeter of the eye may also be a useful metric, or normalizing against a non-ocular dimension, such as total larval length.
- violin plots may also be useful to graph the same data to highlight not only changes in mean values, but also in total distribution and skews in distribution.
- could the authors comment on the significance of reduced OKR and contrast detection? Does this correspond to (for example) reduced efficiency of photoreceptor function, or second-order transmission, or of brain processing? This may not be completely known, but it would be nice to speculate, I think.
- the authors speculate on the role of cathepsin D in outer segment phagocytosis and its potential reduction following CSE exposure. Have they considered measuring expression levels of cathepsin D under this treatment regime?

Points for clarification:

- are the fli1:eGFP fish on the same background as the wild-type?
- are the larvae acclimated before the start of OKR?
- are fish anesthetized before culling with PFA?
- "their diameter measured using arbitrary lines generated with OLYMPUS cellSens Standard software" - I'm sure the scaling was done correctly, but it sounds like the branch diameter is being measured arbitrarily.
- for white light histological analysis, do the authors have a way to standardized a central plane for analysis - optic nerve, or other landmark?
- "uninflated swim bladders are often observed in zebrafish with impaired vision" - and other defects also. Have the authors considered selecting only treated larvae with inflated swim bladders to reduce the confound of swim-bladder inflation from behavioral response based on locomotion?

Is the study design appropriate and does the work have academic merit?

Yes

Is the work clearly and accurately presented and does it cite the current literature?

Yes

If applicable, is the statistical analysis and its interpretation appropriate?

Yes

Are all the source data underlying the results available to ensure full reproducibility?

Yes

Are the conclusions drawn adequately supported by the results?

Yes

Are sufficient details of methods and analysis provided to allow replication by others?

Yes

Reviewer Expertise:

Animal models, retinal development, retinal disease, inherited eye disease

I confirm that I have read this submission and believe that I have an appropriate level of expertise to confirm that it is of an acceptable scientific standard, however I have significant reservations, as outlined above.

# Response

**Published:** 2023-08-11

**Authors:** Alicia Gómez Sánchez

**Affiliations:** 22032, Ireland

Dear Dr. Collery, We greatly appreciate your constructive review of our work. Your insightful suggestions for improvement are highly appreciated. Below, we provide responses to your individual comments.

Minor points:

 1. Though the means of the 0.06 cpd OKR test were not statistically significant, the variances seem to be. The authors could consider using an F-test to look for differences in variance for 0.06 cpd groups (e.g. Levene Test ( https://www.itl.nist.gov/div898/handbook/eda/section3/eda35a.htm)

**Response: **We appreciate this input about our statistical analysis. We performed an F-test, specifically, the Levene Test that the reviewer suggested. The analysis revealed that the variance across 0.06 cpd groups is not statistically significantly different (p=0.77). The Figure 2 legend has been updated to state “A Levene's test was performed and the variance across 0.06 cpd groups is not statistically significantly different (p=0.77)”.

 2. Could the fli1:eGFP line identify differences in brain vasculature after CSE exposure that might suggest a mechanism for altered behavior?

**Response: **Thank you for the insightful question. We agree it would be an interesting future experiment to assess the effects of CSE on brain vasculature using the fli1:eGFP line and look for further associations to behavioural changes. However, in the context of the visual behaviour changes reported in this manuscript we note a recent publication shows NGF-loaded nanoparticles injected in the zebrafish eye can significantly rescue the OKR defects in our CSE model ^1^ (see references at the end). This suggests the eye is a major player in the decreased OKR behaviour.  

 3. Are genes other than cat, gpx1a, nfe2l2a altered? RNAseq analysis might highlight pathways affected, including ones not considered by the authors.

**Response: **We thank the reviewer for this interesting suggestion. In our study, we were only able to assess the genes shown. We hope that we or others can build upon this finding and apply genomic-based approaches to analyse further genes, but this is subject to future availability of funding and resources.  

 4. Would zebrafish allowed to recover to juvenile/adult status after CSE continue to show differences in retinal vasculature or behavior? This may be challenging from an AREC standpoint but could be justified by the importance of understanding the effects of cigarette smoke exposure during early development on human vision and mental health in later life.

**Response: **We agree that this is an interesting experiment and one we discussed internally. As the reviewer mentions, a rate-limiting step here is the ethical approval required to conduct these experiments. Therefore, we are currently not in a position to perform the experiment, but we hope that publication of the manuscript will enable us or others in the community to do so in the future.  

 5. Could the authors comment on the degree of contrast between the different colors in the drums e.g. black vs white seems to have higher contrast than black and red, or black and blue. Does this assay truly test chromatic detection limits or contrast sensitivity?

**Response: **We were particularly careful in the manuscript to state that we were assessing visual behaviour in response to drums with the particular colour patterns shown. This builds upon a previous publication using similar black/colour patterns ^2^. We do not conclude that these truly reflect solely chromatic detection limits or contrast, indeed the response is likely an integration of both. To directly address the question of the degree of contrast we analysed the Web Content Accessibility Guidelines (WCAG) resource and analysed the contrast ratios for the black-white (B-W); black-red (B-R), black-green (B-G) and black-blue (B-B) drums using the specific RGB codes used to print the 3D-drums ^3,4,5^. The WCAG ratios for our coloured drums are the following: B-W: 21/1; B-R: 5.25/1; B-G:15/3; and B-B: 2.44/1. The B-B contrast is indeed the lowest contrast tested and the one which evokes the least OKR saccades from the fish. At this time, we cannot pinpoint this reduced visual behaviour to colour or contrast alone.  

 6. Could the authors speculate on what is causing the increase in axial length of the larval eye after CSE exposure? The lens size appears to be the likely driver, but the sum of all retinal layers may also meet statistical significance criteria. Measuring the perimeter of the eye may also be a useful metric, or normalizing against a non-ocular dimension, such as total larval length.

**Response:** This is an interesting question about biometry analysis. We agree that the lens appears to be the driver for the increase in axial length since according to our updated analysis, the lens is the only statistically significant feature affected by CSE, with an 8.28% increase with respect to the 0.05% DMSO control group. As the reviewer suggested, we summed all the retinal layers of each larva for both 0.05% DMSO control and 20 µg/ml CSE-treated groups to discern differences in the retina compare with the initial measurements shown in Figure 7B where axial length was measured directly from the apex of the lens to the back of the eye (see legend of Figure 7 for more information). Interestingly, analysis revealed that retinal layers of 20 µg/ml CSE-treated larvae are significantly thicker (96.46 µm; p=0.0039) than 0.05% DMSO control group (87.23 µm). When examining the macroscopic images of the larvae (as shown in Figure 1B), no discernible differences in larval size were observed between 20 µg/ml CSE-treated and 0.05% DMSO control group.  

 7. Violin plots may also be useful to graph the same data to highlight not only changes in mean values, but also in total distribution and skews in distribution.

**Response:** We appreciate the reviewer’s observation. Please see updated Figure 7C where we have included violin plots.   

 8. Could the authors comment on the significance of reduced OKR and contrast detection? Does this correspond to (for example) reduced efficiency of photoreceptor function, or second-order transmission, or of brain processing? This may not be completely known, but it would be nice to speculate, I think.

**Response:** This is a very interesting question, but not simple to answer. Visual behaviour requires the integration of multiple cell types in the eye and the brain. Consequently, a defect in any of these cells or structures can trigger a vision impairment. The underlying onset of reduced OKR in our CSE model may be traced to various areas along this pathway where we have found changes. We could attribute reduced OKR to the lens which is larger in 20 µg/ml CSE-treated larvae. Cigarette smoking accelerates the onset of nuclear cataracts in mouse models, characterized by higher lens thickness ^6^. Our results suggest that an early onset of “cataract-like” conditions may be occurring in the lens, leading to a vision defect and subsequently a decrease in OKR. We also identified that the hyaloid vessels attached to the lens are dilated. Vasculature dysfunction can also affect the OKR as shown previously in a zebrafish model of ocular vasculature abnormalities ^7^. Additionally, at the back of the eye, we found an increased number of phagosomes, implying dysfunction either in the outer segment of photoreceptors or in the RPE due to a difficulty to digest phagosomes, a process crucial for vision.   

 9. The authors speculate on the role of cathepsin D in outer segment phagocytosis and its potential reduction following CSE exposure. Have they considered measuring expression levels of cathepsin D under this treatment regime?

**Response: **Thank you for your interesting question. Previous study ^8^ showed how zebrafish morpholino knockdown of cathepsin D retinae were reduced in size with RPE cells lacking microvilli. Therefore, it would be of interest to assess the levels of cathepsin D in the larval eyes following CSE exposure, as this would provide preliminary insight on the stage of the OSP process being affected by CSE. These experiments, however, are contingent upon the availability of resources such as antibodies specific for zebrafish cathepsin D, which we currently do not have.  

Points for clarification:

 a. are the fli1:eGFP fish on the same background as the wild-type?

**Response: **Yes, they are.  

 b. are the larvae acclimated before the start of OKR?

**Response: **The methylcellulose (mixed with embryo medium) used for immobilizing zebrafish in the OKR experiment is warmed up for 20 min at 27° in the incubator to prevent drastic temperature changes in the larvae. Prior to starting the OKR, larvae are kept in the incubator to a peak light wavelength (λp) of 547 nm and a temperature of 27°. Immediately before starting OKR experiments, larvae are transferred to the analysis room within the fish facility, where the parameters are similar to the incubator: a peak light wavelength (λp) of 601 nm and a temperature of 28°.  

 c. are fish anesthetized before culling with PFA?

**Response: **No, they are <131 hpf so anesthesia is not required before fixation in PFA.

 d. "their diameter measured using arbitrary lines generated with OLYMPUS cellSens Standard software" - I'm sure the scaling was done correctly, but it sounds like the branch diameter is being measured arbitrarily.

**Response: **We appreciate the reviewer’s observation. Branch diameter was indeed consistently measured right after hyaloid artery branches out from the optic disc. We have corrected our original state ‘The number of main branches radiating from the optic disc area was quantified and their diameter measured using arbitrary lines generated with OLYMPUS cellSens Standard software’ to ‘The number of main branches radiating from the optic disc area was quantified and their diameter was measured by drawing a perpendicular line to the wall of the vessel, right after the point of branching out from the hyaloid artery, using the “arbitrary line” tool in OLYMPUS cellSens Standard software”.   

 e. for white light histological analysis, do the authors have a way to standardize a central plane for analysis - optic nerve, or other landmark?

**Response:** Only sections where the optic nerve was visible were chosen for analysis. For measurement purposes, a straight line was traced linking both marginal zones and from that line, a perpendicular line from the apex of the lens to the back of the eye was drawn. AL and Lens thickness were taken on this line.  

 f. "uninflated swim bladders are often observed in zebrafish with impaired vision" - and other defects also. Have the authors considered selecting only treated larvae with inflated swim bladders to reduce the confound of swim-bladder inflation from behavioral response based on locomotion?

**Response: **We thank the reviewer for this point. We did not remove the fish with uninflated swim bladders (but otherwise normal gross morphology) from the behavioural assays. This is a phenotype of the CSE treatment and therefore the reported behaviour reflects the overall outcomes. Notably, the OKR does not require locomotion and the visual behaviour defects reported are not confounded. It is unclear whether swim bladder inflation is needed for the VMR assay, as this is performed in shallow multi-well plates and the larvae do not need to change depths. Indeed, incompletely inflated swim bladders are not needed for locomotion as shown in Fig 1, where although ~40% of larvae show incompletely inflated swim bladders, less than 10% show an absent startle locomotor response.  

 References:

 1. Colucci P, Giannaccini M, Baggiani M, Kennedy BN, Dente L, Raffa V, Gabellini C. Neuroprotective Nanoparticles Targeting the Retina: A Polymeric Platform for Ocular Drug Delivery Applications. Pharmaceutics. 2023; 15(4):1096.

 2. Deveau C, Jiao X, Suzuki SC, Krishnakumar A, Yoshimatsu T, Hejtmancik JF, et al. (2020) Thyroid hormone receptor beta mutations alter photoreceptor development and function in Danio rerio (zebrafish). PLoS Genet 16(6): e1008869.

 3. AccesibleWeb. (2023). Color Contrast Checker. Retrieved from https://accessibleweb.com/color-contrast-checker/

 4. WebAIM. (2023). Colour Contrast Checker. Retrieved from https://webaim.org/resources/contrastchecker/

 5. Adobe. (2023). Color Contrast Analyzer. Retrieved from https://color.adobe.com/es/create/color-contrast-analyzer

 6. Avunduk AM, Yardimci S, Avunduk MC, Kurnaz L, Koçkar MC. Determinations of some trace and heavy metals in rat lenses after tobacco smoke exposure and their relationships to lens injury. Exp Eye Res. 1997 Sep;65(3):417-23. doi: 10.1006/exer.1997.0344. PMID: 9299178.

 7. Ward R, Ali Z, Slater K, Reynolds AL, Jensen LD, Kennedy BN. Pharmacological restoration of visual function in a zebrafish model of von-Hippel Lindau disease. Dev Biol. 2020 Jan 15;457(2):226-234. doi: 10.1016/j.ydbio.2019.02.008. Epub 2019 Feb 27. PMID: 30825427

 8. Follo, C. et al. Knock-down of cathepsin D affects the retinal pigment epithelium, impairs swim-bladder ontogenesis and causes premature death in zebrafish. PLoS One 6, e21908 (2011)
